# Transanal access port (TrAAP) technique: the use of a single incision laparoscopic surgical port during canine colonoscopy (a cadaveric study)

**DOI:** 10.1186/s12917-021-02753-9

**Published:** 2021-01-21

**Authors:** James Howard, Judith Bertran, Valerie Parker, Jenessa Winston, Adam J. Rudinsky

**Affiliations:** 1 The Department of Veterinary Clinical Sciences, College of Veterinary Medicine, The Ohio State University, 601 Vernon L. Tharp St, Columbus, OH 43210 USA; 2 Comparative Hepatobiliary and Intestinal Research Program (CHIRP), College of Veterinary Medicine, The Ohio State University, 601 Vernon L. Tharp St, Columbus, OH 43210 USA; 3 The Department of Small Animal Clinical Sciences, College of Veterinary Medicine, The University of Florida, 2015 SW 16th Ave, PO Box 100126, Gainesville, FL 32610-0126 USA

**Keywords:** SILS, Single-incision laparoscopic surgical port, Colonoscopy, Endoscopy, Minimally-invasive

## Abstract

**Background:**

Transanal colonoscopy using the single-incision laparoscopic surgical port is routinely used in human patients but has not been described in veterinary literature. The purpose of this study was to describe a novel access technique elucidating its endoscopic clinical potential and benefits. Additionally, its challenges, limitations, and clinical usability will be discussed and critiqued. The aim of this study was to describe the feasibility of the single-incision laparoscopic surgical port (SILS) as a transanal access technique in canine cadavers and compare its technical capabilities and economic value when compared to the traditional approaches of digital pressure and purse string.

**Results:**

The overall time to reach an intraluminal pressure of 10 mmHg was faster for digital pressure versus purse string (*p* = 0.05) and faster for single-incision laparoscopic surgical port versus purse string (*p* < 0.02). Maximum luminal pressure was significantly higher between single-incision laparoscopic surgical port and purse string (*p* = 0.001). Mean pressure for both the complete 60 s trial and during the last 45 s of insufflation were highest with the SILS port and were significantly different between the single-incision laparoscopic surgical port versus purse string (*p* = 0.0001, *p* < 0.0001) and digital pressure versus purse string (*p* < 0.005, *p* < 0.01) respectively. Complete luminal distention and visualization was observed in all trials.

**Conclusions:**

The SILS port in a cadaveric canine model allowed good visualization of the rectal and colonic mucosa, provided constant insufflation of the colon and was feasible and subjectively easy to perform. Technical differences between techniques were observed with the use of the SILS port allowing for potentially lower personnel requirements, less procedural associated cost, less variability versus the digital pressure technique between assistants, and the ability of additional instruments to be used for procedures.

## Background

Colonoscopy is a frequently utilized diagnostic modality for the evaluation of canine intestinal diseases. This minimally invasive technique provides the diagnostician optimal visualization of mucosal surfaces with concurrent ability to obtain partial thickness biopsies reducing the incidence and necessity for more invasive surgical techniques [1]. Full thickness colonic biopsies are uncommon; therefore, the surgical diagnostician will often collaborate with endoscopists to obtain complete intestinal biopsy profiles. In certain cases surgical removal of mass lesions (benign and malignant) become necessary for palliation of clinical signs or representative sampling for diagnoses. Unobstructed visualization is imperative in order to provide a safe passageway through the lower intestinal tract resulting in appropriate evaluation of the entire rectal, colonic, and ileal mucosal surfaces for sampling. To achieve optimal visualization, insufflation and distention of the intestinal tract lumen is necessary [2]. It has been recommended that circumferential digital pressure be applied around the scope using the rectum as a seal to assist with insufflation and to prevent air leakage and luminal collapse during the procedure [3, 4]. If insufflation assistance is not performed appropriately, it can result in decreased visualization, poor endoscopic maneuverability, increased procedural time, and require additional procedural personnel.

The single-incision laparoscopic surgery (SILS) port is a port with multiple access cannulas with one designated for insufflation. This port is designed for use in human laparoscopic surgery and transanal endoscopic microsurgeries. It is described for use in veterinary surgery, including but not limited to, uterine and ovarian procedures, laparoscopic assisted gastropexy, and small intestinal foreign material. With increased usage among surgeons, and the ever-present economic incentive to minimize consumable goods, a recent publication described the SILS port sterilization and re-sterilization properties with an emphasis on bacteriological scores [5]. It is composed of a highly flexible soft thermoplastic elastomer allowing for safe and atraumatic transanal access [6]. The purpose of the port is to provide procedural maneuverability through three depth-adjustable baffled cannulas thereby improving visualization and instrument manipulation while maintaining adequate insufflation pressures. The instrument ports fit routine laparoscopic and endoscope cannula sizes ranging from 5, 10, 12, and 15 mm, and the adjustable insufflation ports control peritoneal or intestinal lumen insufflation pressures with minimal effort. The versatility of the SILS port facilitates a myriad of minimally invasive surgical procedures depending on its application in people. This allows for minimally invasive colonic surgical procedures thereby decreasing the risks associated with the terminal portion of the intestinal tract. To the authors’ knowledge, objective colonic and rectal accessibility coupled with a visualization scoring system using a transanal approach with the SILS port in canine patients has not been evaluated.

The purpose of this feasibility study was to demonstrate the utility of the SILS port as a transanal access port (TrAAP) for the use of endoscopic colonic procedures in canine cadavers. Based on the use of the SILS port for other routine minimally invasive surgical procedures, coupled with the known challenges facing colonoscopy, we hypothesized that the SILS port would provide appropriate visualization of rectal tissues as well as adequate distention of rectal and colonic lumens. If successful, the SILS port can be further investigated for continued procedural research including: objective assessments of variable endoscopic instrumentation, objective intraluminal workability and field-of-view, live patient safety evaluations for colonoscopy, rectal surgical procedures, other clinical trans-anal visualization needs and its ability to improve procedural efficiency.

## Results

Time to reach 10 mmHg luminal pressure for DP [median 6 s (range 3–12)], PS [median 10 s (range 3–60)], and SILS [median 3.5 s (range 3–10)] are reported. A significantly shorter duration to reach 10 mmHg was observed with DP versus PS (*p* = 0.05) and a significantly shorter duration to reach 10 mmHg was observed using the SILS port versus PS (*p* < 0.02) (2A). There was no significant difference in the time to reach the luminal pressure of 10 mmHg when comparing DP versus SILS (*p* = 0.8).

**Fig. 1:**
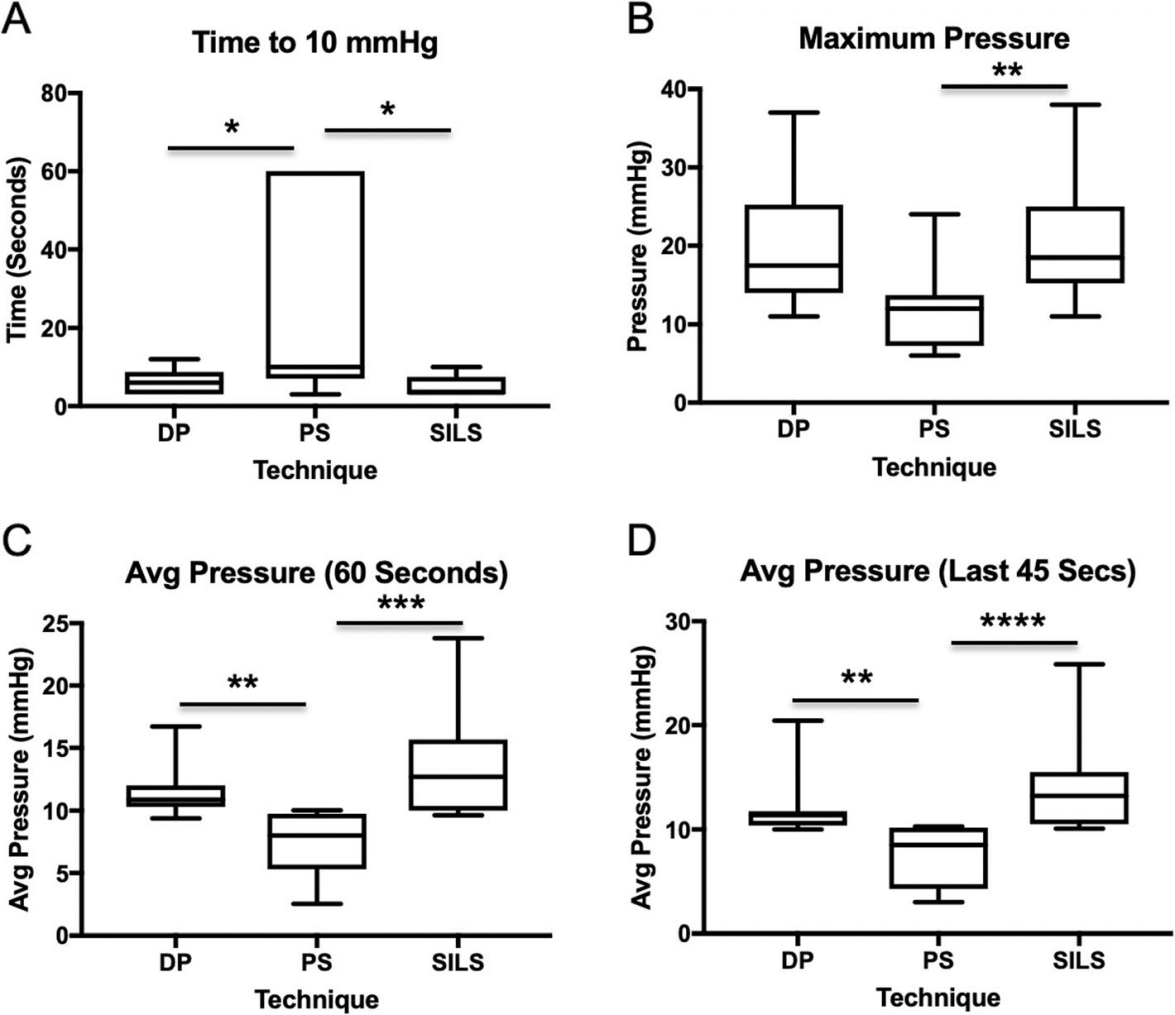
Insufflation variables assessed during study: **a** Time to reach 10 mmHg luminal pressure, **b** Time to maximum pressure, **c** Average pressure during complete 60 s study, **d** Average pressure during final 45 s of the study (stable period)

Maximum luminal pressure (mmHg) achieved for DP [median 17.5 mmHg (range 11–37)], PS [median 12 mmHg (range 6–24)], and SILS [median 18.5 mmHg (range 11–38)] are reported. Maximum luminal pressure differed significantly between techniques (*p* = 0.001). Significantly different maximum luminal pressures were noted between SILS and PS (*p* = 0.001) techniques (Fig. 1b). There was no difference in maximum pressure between DP and SILS (*p* = 0.55) or DP and PS (*p* = 0.07).

Average luminal pressure for the complete 60 s of the trial for DP [median 10.88 mmHg (range 9.38–16.72)], PS [median 7.99 mmHg (range 2.5–10)], and SILS [median 12.72 mmHg (range 9.63–23.8)] are reported. Average luminal pressure over 60 s differed significantly between time points (*p* < 0.0001). Significantly higher average pressures were measured with the SILS versus the PS (*p* = 0.0001) and with DP versus the PS (*p* < 0.005) techniques (Fig. 1c). There was no difference in average pressure between DP and SILS (*p* = 0.99) over the complete 60 s trial. There were no differences in the variances between groups (*p* = 0.21).

Average luminal pressure for the final 45 s of the trial for DP [median 11.38 mmHg (range 10.02–20.44)], PS [median 8.5 mmHg (range 3–10.29)], and SILS [median 13.24 mmHg (range 10.09–25.87)] are reported. Average pressure during the final 45 s of the trial differed significantly between time points (*p* < 0.001). Significantly higher average pressures were measured with the SILS versus the PS (*p* < 0.0001) and higher with DP versus the PS (*p* < 0.01) techniques (Fig. 1d). There was no difference in average pressure between DP and SILS (*p* = 0.66). There were no differences in the variances between groups (*p* = 0.29).

Luminal distension of the rectum and distal colon was evaluated on a semi-subjective scale. In all dogs for all three trials complete luminal distension and visualization was achieved. Further statistical testing was not performed as all groups achieved complete distension.

## Discussion

Despite the widespread use of colonoscopy as a diagnostic modality, transanal access techniques have undergone minimal evaluation to maximize personnel, cost savings, ease of use, and efficiency. As with any new procedure, it must be safe for the patient, cost effective, and have a learning curve in which proficiency of the procedure can be reasonably accomplished by those performing the task [7]. As a proof-of-concept, the TrAAP procedure was evaluated as an alternative to more traditional colonoscopy access techniques and found to have multiple potentially clinical benefits.

The performance of the SILS technique was comparable to the traditional assisted insufflation approach using DP. This preliminary data provides evidence that the SILS should be utilized in additional clinical studies in live research animals and potentially clinical patients to better assess its safety and diagnostic utility. This is essential as the main benefits of the SILS are that it 1) provided visually consistent clinical insufflation with minimal handling of the perianal region, and 2) easy visualization free of any obstruction of the targeted intestinal tissue regions, including visualization of rectal tissues without necessitating retroflexion of the endoscope.

Furthermore, as veterinary overhead costs and hourly wages continue to increase, the SILS port can provide cost savings to the practitioner while allowing for a more efficient and appropriate use of their technical staff. For example, given the most recent evidence for re-sterilization options using the SILS port, 10 uses are acceptable [5]. When SILS port use is compared to the purse string technique, taking into account the average market cost of suture packs and cost and re-sterilization of the SILS port over 10 procedures, a savings of approximately $100 is achieved by using the SILS port. Additionally, when comparing the SILS port cost and usage versus the nationally hourly wage of a technician providing digital pressure for 10 procedures, the cost savings are approximately $130 [8]. Therefore, as the SILS performed comparably to DP, utilization of the SILS enables elimination of the personnel requirement for the DP technique. This translates to a cost-effective, personnel minimizing, and efficient modality to perform lower intestinal endoscopy for the practitioner.

The endoscopic access through the SILS port was established by removing the designated insufflation tubing and replacing the endoscope in its place. This was done to standardize the insufflation process for all three groups using the red rubber insufflation catheter. In clinical practice, using a traditional cannula to accommodate the scope size along with insufflation through the designed tubing, may be a superior choice allowing an even greater ability to manipulate the endoscope throughout the procedure. Overall, the SILS Port was effective in the cadaveric model used here and has the potential to greatly improve canine colonoscopy cost structure, efficiency and comprehensiveness in clinical practice.

Contrary to the hypothesis of this study, the results failed to identify a significant difference between the SILS and DP techniques. In this study, DP was applied by a single individual who was particularly well adept at providing consistent DP. In practice, the individuals administering DP may have varying skill levels and consistency of performance, and as a result may provide more variable results than those reported here. Further studies are needed to examine variability within and between DP individuals as well as a more specific examination of differences in patient comfort between the two procedures as one procedure may be better suited for canine endoscopy than the other.

Our study included dogs limited to those weighing 15 kg or greater. The SILS port is highly compressible to approximately 60% of its original diameter with an overall length of 50 mm, maximum outer diameter of 50 mm, and inner diameter of 30 mm [9]. However, the feasibility and usage of this device in smaller patients (< 15 kg) with smaller diameter anuses remains to be investigated. Additionally, its effect on discomfort, distensibility, and operator ease in dogs with minimally compliant anuses or increased anal tone was also not evaluated and remains speculative at this stage.

There was no difference in the ability of the three techniques to visualize and distend the rectum and distal colon based on visual assessments. Despite good static visualization and distension being achieved, further studies are necessary to compare the procedural maneuverability between these techniques. Subjectively, the manipulation of the endoscope with both DP and PS were more likely to result in air leakage and loss of insufflation than when done with SILS. This air leakage would likely be worse if the procedure required transanal passage of equipment other than the endoscope this would likely be worsened. The TrAAP technique may mitigate some of these procedural issues. Additionally, availability of multiple sized cannulas (5 mm, 10 mm, 12 mm, and 15 mm) allows for wide variability of instrument and endoscope selection. The SILS port has two additional ports not used during this experiment; this allows two additional instruments to be inserted for interventions while also maintaining the insufflation and seal. This is a major benefit to the SILS compared to other techniques, especially when performing endoscopically guided sampling and surgical procedures.

The PS procedure was used as a separate control for an additional personnel independent technique to assist with insufflation other than SILS. This technique was chosen as it relies on equipment routinely available in clinical practice, as well as a simple technique that is manageable by most veterinarians. Unfortunately, the PS technique failed to consistently perform in a manner comparable to either the current standard DP or novel approach with the SILS port. Although the distention/visualization was adequate, the insufflation pressure was showed to be inferior. Additionally, although not directly measured, the rate of leakage was noticeably higher in this technique that could result in clinician frustration and loss of insufflation and visualization in more prolonged procedures. Further testing of the PS approach to provide better insufflation and visualization is not recommended based on these results, time required to place the purse string suture, and risks associated with purse string sutures such as anal sac penetration or residual suture left behind following attempted removal.

Limitations of this study relate to the quantification of the insufflation gas and the technique for its introduction into the rectum. First, the amount of carbon dioxide (CO2) used to reach 10 mmHg, or to maintain this pressure for a predetermined amount of time, was not quantified. The reason for this lack of data was due to the inability to objectively standardize measuring the amount of CO2. Carbon dioxide escaping from the anus with each technique (SILS, DP, PS) versus traveling proximally within the colon and into the cecum, ileum, and other bowel segments, could not be accurately accounted for in the cadaveric model. The authors were concerned with cadaveric variability regarding ileocecal colonic junction patency thus allowing variable amounts of air to escape into the intestinal tract in an oral direction. Presumptively, this “leakage” could have been attributed to the specific technique used (SILS, DP, PS), when instead it was due to continued intestinal insufflation. Further research is needed to investigate specific gas usage to insufflate the rectum and colon. Second, the insufflation port provided standard with each SILS port was not used; instead a red rubber catheter was utilized. This was done to standardize the rate and amount of CO2 provided with each technique. The insufflation tubing provided with the manufacturer’s SILS port could not be effectively used due to its short length during the digital pressure or purse string portions of the experiment, thus introducing variability into the experimental design and outcomes. Further research is being conducted to evaluate the efficacy of standard insufflation ports in live animals.

Another limitation of this study was the lack of intestinal imaging modalities. Radiographic interpretation could potentially have elucidated if ileocecocolic valve insufficiency existed. However, it was cost prohibitive at the authors institution; this is being evaluated in subsequent clinical patient evaluations. Additionally, the use of a 30 degree angle endoscope or Endocameleon was not used. The authors designed the study for both rigid or flexible forward facing (0 degree) endoscopes to accurately visualize and assess intraluminal distension to provide acceptable visualization. Visualization in this study was defined as the ability to see all surfaces without mucosal folding. In future projects, visualization defined as ability to visualize different surfaces with different endoscopes should be examined. This is especially important with continued research into procedural techniques requiring different viewing angles using a variety of endoscopes.

Finally, further limitations of the present study were the inability to examine factors such as patient comfort, injury and/or stress to rectal sphincter tissue, sphincter function following the procedure, ideal insufflation rates, or ideal insufflation pressures to appropriately minimize patient discomfort and risk while maximizing diagnostic benefit. Additionally, another limitation of this study was the lack of a standardized sham procedure reflecting motions and manipulations common during this a colonoscopy procedure. However, these concerns and questions should be addressed in a live animal model with a different study design. Further investigation of the TrAAP technique is warranted to investigate those variables and then continued into a clinical model to determine the utility of this procedure for minimally invasive surgical techniques.

## Conclusion

In conclusion, the TrAAP technique using the SILS port in a cadaveric canine model allowed good visualization of the rectal and colonic mucosa, provided constant insufflation of the colon and was feasible. Although these results were comparable to the standard technique using digital pressure to achieve a seal for colonoscopy, the use of the SILS port for that application may bring the advantage of less needed personnel, less procedure associated cost, less variability in DP technique between assistants, and most importantly, the ability of additional instruments to be used for interventional procedures of the rectum and colon.

## Methods

Eighteen canine cadavers were acquired after being euthanized at a local shelter for reasons unrelated to the study in accordance with the American Veterinary Medical Association’s *Guidelines for the Euthanasia of Animals* [10]. Dogs were required to be at least 15 kg of body weight and have normal perianal structures on physical examination. The study protocol was approved by the Clinical Research Committee at the authors’ institution. Cadavers were stored at 2 °C for up to 48 h prior to the study. All cadavers were allowed to equilibrate to room temperature prior to beginning the experiment. The experiment was a randomized crossover design. Each cadaver was randomly assigned to one of three groups. Each group then underwent all three colonic distension techniques: Digital Pressure (DP), Purse String Suture (PS), and SILS port (SILS; Medtronic, Minneapolis, MN). Group 1 had the DP technique performed first, followed by PS, and then followed by SILS. Group 2 had the PS technique performed first, followed by SILS, and then followed by DP. Group 3 had the SILS technique performed first, followed by DP, and then followed by PS.

The cadavers were positioned in left lateral recumbency for all procedures. A pneumocolon was accomplished in two different manners depending on the group. For the PS and DP groups an Olympus GIP-P140 9.8 mm endoscope and a 16 French (5 mm diameter) red rubber pressure catheter was placed in the rectum 3 cm past the external anal sphincter. For these techniques Arthrex insufflation tubing with an Arthrex Synergy Insufflation AR-3290-0004 set (Arthrex Naples, FL) was attached to the red rubber pressure catheter using a Christmas tree adapter; maintenance of 10 mmHg intraluminal pressure with a flow rate of 3 L/minute was performed based on clinical experience and its similarity to human literature. Previous studies in the human literature use approximately 13 mmHg to provide adequate insufflation [6].

**Fig. 2:**
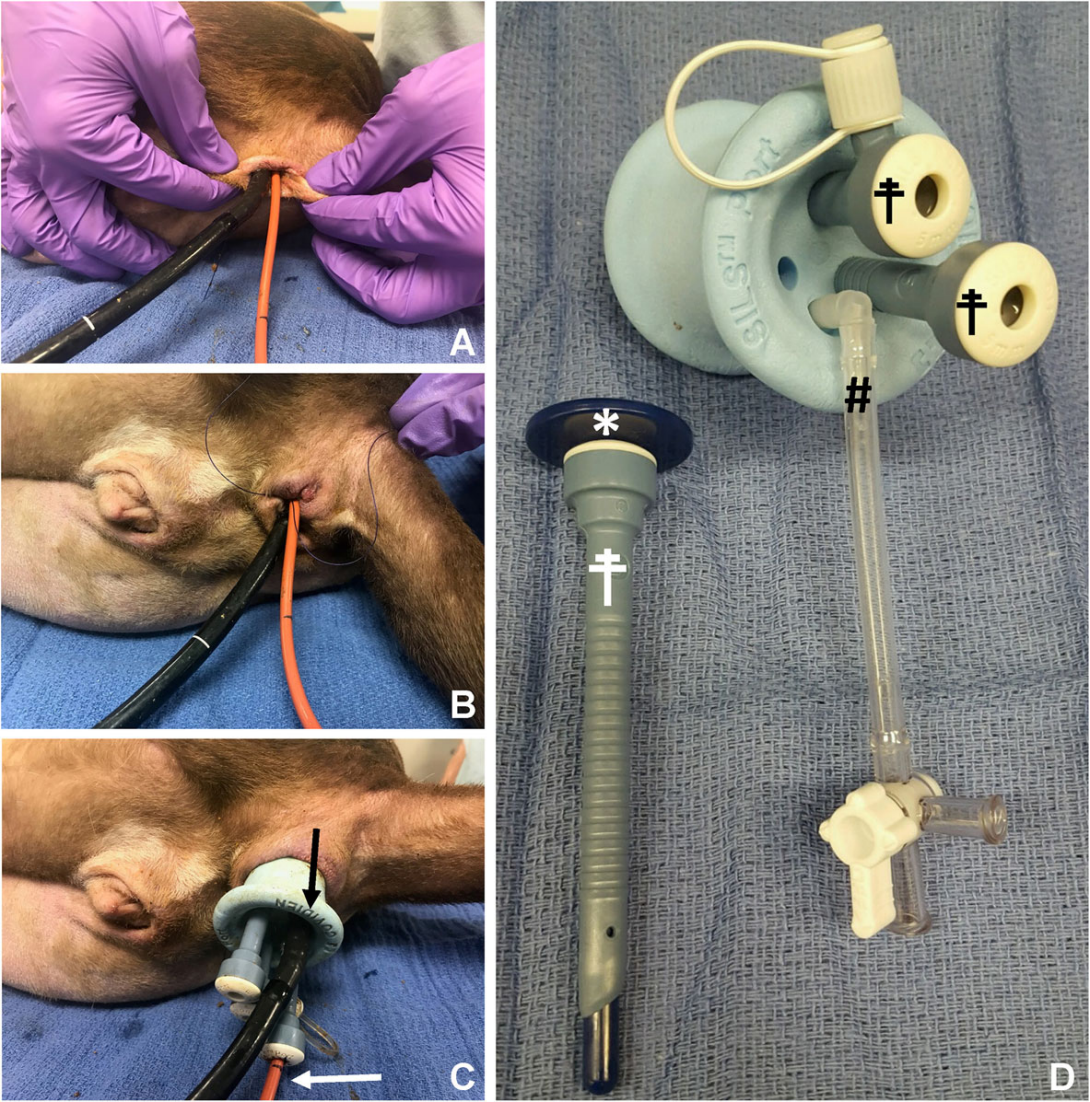
Representative images of the 3 insufflation techniques: **a** Digital pressure (DP) technique: Notice the firm circumferential pressure with elliptical deformation of the anus creating a working seal. **b** Purse string (PS) suture technique: Notice the centripetal accordion deformation of the anus due to suture passage along the outer rim of the recto-anal junction. Suture tags are left long to facilitate removal. **c** TrAAP technique using the SILS Port: Notice that all three cannulas are in place with the designated insufflation tubing removed (see # in D section); the endoscope was inserted into its place. The red rubber insufflation catheter was inserted into a traditional cannula to establish the pneumocolon. **d** SILS Port anatomy: Note the clear designated insufflation tubing with a provided thee-way-stop cock (#). This was removed to facilitate endoscopic passage in this study. Three separate depth adjustable instrument ports are provided (☨). Each cannula is inserted into the SILS Port using the provided trocar (*). The SILS Port accommodates 5 mm (shown here - ☨), 10 mm, 12 mm, and 15 mm cannulas allowing for various instrumentation and optics

For the SILS evaluation a SILS Port was lubricated and manually advanced into the rectum ensuring that the internal rim was seated within the rectal tissues. Once insertion into the rectal vault was observed, the device was checked for pull-out with gentle caudal traction. Next, the three baffled cannulas were placed into the portal holes with the provided obturators. Finally, the Arthrex Synergy Insufflation was attached to the red rubber pressure catheter as previously described, inserted into a cannula, and the pneumocolon was established. Maintenance of the pneumocolon was achieved with a targeted intraluminal pressure of 10 mmHg and an automatically adjustable flow rate initially set at 3 L/minute. All procedures were started after the rectum and distal colon were manually evacuated of air and feces and no mechanical insufflation occurring. A neutral pressure reading of 0 mmHg was achieved in each cadaver prior to beginning the insufflation process. Pressure recordings were monitored continuously and recorded every second for 60 s regardless of the maximum pressure achieved prior to this endpoint. No measurements were recorded beyond 60 s and no concurrent sham procedures were attempted during this time. To insert the endoscope, the factory provided insufflation tubing (Fig. 2) included with each SILS port was removed and the endoscope was inserted in its place. This was done to keep the insufflation technique consistent amongst all techniques. The diameter of the factory insufflation tubing is identical to the other cannulas and in a clinical patient a cannula can be removed for endoscope insertion and the provided insufflation tubing can be used. Visualization of the rectal and colonic lumen was continuously recorded throughout the procedure and analyzed thereafter; sections of the intestinal tract orad to the colon (cecum, ileum, jejenum, duodenum) were not included in the investigation.

Rectal seals to maintain an appropriate level of insufflation were evaluated for each group. DP was applied by one author using two thumbs and two index fingers on either side of the scope and catheter (Fig. 2a). A perianal purse string (PS) suture was placed circumferentially around the anus as previously described in routine fashion to ensure a subjective tight seal around the instrumentation (Fig. 2b) [11]. The cannulated SILS Port (Fig. 2c) was placed in the rectum as described previously. One cannula was occupied by the red rubber pressure catheter attached to insufflation; two cannulas were left empty; and the designated insufflation access tubing was removed from the port to accommodate the 9.8 mm endoscopic camera inserted into its place with the use of surgical lubrication. The camera was large enough to create an airtight seal while allowing forward and backward movement of the scope.

Variables of interest in the statistical analyses included effect of technique on time to reach 10 mmHg luminal pressure, maximum luminal pressure, average pressure over complete study (60 s), and average pressure over last 45 s (defined stable period as each cadaver took less than 15 s to reach a steady 10 mmHg insufflation state). Colonic and rectal distension and visualization were categorized by one author with experience in colonoscopy as no distension/visualization (less than 25% diameter distension), partial distension/visualization (26–75% diameter distension), or complete distension/visualization (greater than 75% diameter distension).

Descriptive statistics were analyzed and variables were tested for their distribution using the Shapiro-Wilk test. Descriptive statistics are reported as median (range). Variables of interest were compared using a Friedman test followed by Dunn’s multiple comparisons test. Variance of pressure readings throughout the study were compared between groups with the Fligner-Kileen test. All statistical analyses were performed using either GraphPad Prism^1^ or R software^2^. Order of testing was assessed for a crossover effect in the randomized repeated measures design and was found to have not impacted results. All analyses were therefore analyzed together without order of testing as a variable in further analyses. Statistical differences were set at a *p* < 0.05.

## Data Availability

The datasets used and/or analysed during the current study are available from the corresponding author on reasonable request.

## Abbreviations

SILS: Single incision laparoscopic surgery
TrAAP: Transanal access port
DP: Digitial pressure
PS: Purse string

## Footnotes

1 GraphPad Prism (La Jolla, California)

2 R Software (The R Foundation)
